# Tripartite motif containing 21 autoimmunity primes CD8^+^ cell–mediated lung inflammation: an in vivo model of interstitial lung disease pathogenesis

**DOI:** 10.1186/s13075-026-03850-6

**Published:** 2026-06-26

**Authors:** Yulu Qiu, Qin Wu, Lu Liu, Wenjuan Zhao, Yue Wang, Fang Wang, Wenfeng Tan

**Affiliations:** 1 Department of Rheumatology, The First Affiliated Hospital of Nanjing Medical University, Nanjing, Jiangsu China; 2 Department of Cardiology, The First Affiliated Hospital of Nanjing Medical University, Nanjing, Jiangsu China

**Keywords:** TRIM21, Interstitial lung disease, Connective tissue diseases, CD8^+^ cells

## Abstract

**Background:**

Anti-Ro52 [Tripartite Motif Containing 21 (TRIM21)] antibodies are strongly associated with interstitial lung disease (ILD) in connective tissue diseases (CTDs), yet the underlying mechanisms remain elusive. This study aimed to elucidate the mechanisms by which TRIM21 autoimmunity drives ILD using an in vivo model.

**Methods:**

A murine model of TRIM21 autoimmunity was established by immunizing mice with full-length recombinant TRIM21 protein. Lung immune infiltration and antibody production were assessed. To mimic viral triggers, mice received poly(I:C). Immune cell subsets and transcriptional changes were analyzed using flow cytometry, RNA sequencing, and immunohistochemistry.

**Results:**

TRIM21 immunization induced systemic autoantibody production and B cell activation, which were associated with selective pulmonary inflammation marked by IgG deposition. Upon poly(I:C) challenge, lung inflammation was markedly exacerbated, with increased infiltration of CD8^+^ cells and CD11b^+^ myeloid cells and enrichment of chemokine signaling pathways. RNA sequencing identified Ccl6 as a top upregulated gene, and its protein expression was spatially validated in inflamed lung tissue.

**Conclusion:**

These findings support a pathogenic role for anti-Ro52/TRIM21 autoimmunity in CTD-associated ILD. Our in vivo model reveals a lung-predominant inflammatory response associated with CD8^+^ cell that is amplified by viral mimicry and accompanied by CCL6 signaling, offering mechanistic insight and potential therapeutic targets in anti-Ro52 positive ILD.

**Supplementary Information:**

The online version contains supplementary material available at https://doi.org/10.1186/s13075-026-03850-6.

## Background

Interstitial lung disease (ILD) is one of the most common complications of connective tissue diseases (CTD), including idiopathic inflammatory myopathies (IIM), systemic sclerosis (SSc), and primary Sjögren’s syndrome (pSS), with reported prevalence ranging from 10% to 70% depending on the disease subtype [1–3]. CTD-associated ILD (CTD-ILD) contributes substantially to disease burden, representing a major cause of morbidity and early mortality in affected patients [4, 5]. Given its high prevalence and clinical impact, early recognition and a deeper understanding of the underlying mechanisms of CTD-ILD are critical for improving patient outcomes.

Among the serological markers associated with CTD-ILD, anti-Ro52 antibodies—which target tripartite motif-containing protein 21 (TRIM21)—are the most frequently detected across multiple CTD subtypes [6–9]. Notably, anti-Ro52 antibodies are present in approximately 30 to 40% of patients with antisynthetase syndrome (ASyS) and up to 40 to 60% of those with anti-MDA5-positive dermatomyositis (DM) —two CTDs characterized by a high prevalence of ILD [10–12]. Clinically, anti-Ro52 positivity correlates with increased ILD risk, disease severity, and poorer outcomes [13]. In particular, the co-expression of anti-MDA5 and anti-Ro52 defines a highly aggressive phenotype prone to rapidly progressive ILD (RP-ILD) [3, 11]. Furthermore, a recent ILD large-scale cohort study further confirmed that anti-Ro52 positivity independently predicts worse progression-free and transplant-free survival [14]. Despite its strong clinical associations, the pathogenic role of TRIM21 autoimmunity in ILD remains poorly defined.

TRIM21 (also known as Ro52) is a cytosolic E3 ubiquitin ligase and a high-affinity Fc receptor, best known for its role in antiviral host defense [15–19]. Under physiological conditions, TRIM21 binds to antibody-coated viral particles that gain access to the cytosol, directing them for ubiquitin-mediated proteasomal degradation—a process that restricts viral replication and clears intracellular immune complexes [20–22]. Beyond its degradative function, TRIM21 also acts as an intracellular immune sensor, triggering downstream signaling cascades such as type I interferon (IFN-I) and NF-κB pathways to amplify innate antiviral responses and maintain immune homeostasis [23, 24].

However, this finely balanced antiviral function may become pathogenic in the context of autoimmunity. TRIM21 is one of the most frequently targeted autoantigens in CTDs, but the functional implications of anti-TRIM21 (anti-Ro52) autoantibodies remain incompletely understood [16].

To address this gap, we established a murine model of TRIM21-directed autoimmunity by immunizing mice with full-length TRIM21 protein, thereby inducing anti-Ro52-like immune responses. To mimic viral exposure and enhance innate immune activation, we administered polyinosinic: polycytidylic acid [poly(I:C)], a synthetic analog of viral double-stranded RNA that activates Toll-like receptor 3 (TLR3). This two-hit model allowed us to investigate how TRIM21 autoimmunity and viral-like stimuli interact in lung inflammation. Our results demonstrate that TRIM21 autoimmunity primes the pulmonary microenvironment, but subsequent poly(I:C) challenge is necessary to trigger robust immune infiltration—particularly involving CD8^+^ cells—and exacerbates chemokine induction, including Ccl6.

Although anti-Ro52 is the most prevalent autoantibody in CTD-ILD, its direct pathogenic role has remained speculative. This study provides the first in vivo evidence supporting a link between TRIM21-directed autoimmunity and immune-mediated lung injury. Our findings demonstrate that TRIM21 autoimmunity, particularly in the context of viral-like innate immune activation, contributes pulmonary inflammation and tissue damage, offering mechanistic insights into the clinical association of anti-Ro52 with progressive CTD-ILD.

## Methods

### Animal model

Full-length mouse TRIM21 protein was synthesized by Novoacine Bio-technology Co., Ltd. (Nanjing, China). Female C57BL/6 (B6) mice, aged 8–10 weeks, were purchased from GemPharmatech (China). Mice were subcutaneously immunized in the back with 100 µg TRIM21 protein emulsified in Complete Freund’s adjuvant (CFA) (Sigma-Aldrich, #F5881) once weekly for four consecutive weeks. The control group mice were immunized with PBS emulsified in CFA following the same protocol. All mice received an intraperitoneal (i.p.) injection of pertussis toxin (PT; 250 ng, APExBIO, #B7273) after the first immunization to enhance the immune response.

On day 23 (24 h after the final immunization), all mice were sacrificed; their blood and tissues (lung, spleen, muscle) were harvested for analysis. In a separate animal experiment set, mice immunized with TRIM21 and PT as described above received an additional intranasal administration of poly(I:C) (4 mg/mL in 50 µL PBS) on day 23 and were sacrificed on day 25.

All experiments were carried out under specific pathogen-free conditions. All animal experiments were approved by the Institutional Animal Care and Use Committee (IACUC) of Nanjing Medical University (Approval id: 22841).

### Coomassie Brilliant Blue (CBB) staining

CBB staining was performed to verify the expression and purity of recombinant murine TRIM21 protein. After sodium dodecyl sulfate-polyacrylamide gel electrophoresis (SDS-PAGE), the gel was rinsed with distilled water. CBB staining solution (Beyotime Biotechnology, #P0003S) was then applied for 30 min. After rinsing, the gels were photographed in a ChemiDoc Imaging System (Bio-Rad, USA).

### TRIM21 antibody detection

Mouse serum samples were analyzed by enzyme-linked immunosorbent assay (ELISA) using plates coated with recombinant Trim21 (2 µg/mL). After blocking with 5% bovine serum albumin (BSA) (Solarbio, #A8020), the plates were incubated with serially diluted serum samples (1:25 to 1:15,625). The optical density at 450 nm (*OD450*) was measured with a microplate reader (BioTek Synergy^2^, USA). Specifically, the antibody titer was defined as the highest serum dilution that yielded an optical density (OD450) value between 1.5 and 2.0 [25]. For group comparisons, we calculated the Geometric Mean Titer (GMT) from the log10-transformed titer data.

### Immune cell isolation and analysis from lung and spleen tissues

Fresh lung and spleen tissues were processed for immune cell isolation. Lung tissues were enzymatically digested with DNase I (50 µg/mL) (Sigma-Aldrich, #DN25) and collagenase type I (1 mg/mL) (Gibco, #17100017) at 37 °C for 45 min, while spleens were mechanically dissociated through 70-µm cell strainers. Erythrocytes were lysed with ACK buffer (New Cell & Molecular Biotech, Suzhou, China, #C100C3).

For spleen B cell isolation, approximately 10⁷ cells per tissue were labeled with anti-CD19 microbeads (Vazyme Biotech, China, #CS202-01) and isolated using magnetic-activated cell sorting (MACS) separation (purity > 95% by post-sort flow cytometry). RNA was extracted from sorted CD19^+^ B cells using the TRIzol method (Accurate Biology, #AG21101) for quantitative real-time PCR (qRT-PCR) analysis of B cell effector molecules. Primers are detailed in Supplementary Table S1.

The remaining cells were incubated with fluorochrome-conjugated antibodies for cell surface staining, including CD4-PerCP (BioLegend, USA, #100537), CD8-FITC (BioLegend, #100706), CD19-FITC (BioLegend, #115506), CD138-PE (ABclonal, China, #A26050), CD11b-FITC (eBioscience, #11-0112-41), F4/80-APC (eBioscience, #17-4801-30). After washing with PBS containing 2% FBS, cells were analyzed on CytoFLEX S flow cytometer (Beckman Coulter, USA).

### Hematoxylin and Eosin (H&E) and masson staining

Right lobes of lung and gastrocnemius muscle tissues were fixed in 4% paraformaldehyde (PFA) for 24 h (lungs) or 48 h (muscle, due to higher tissue density). Tissues were then paraffin-embedded, sectioned at 5 μm thickness, and mounted on slides. For H&E staining, sections were deparaffinized in xylene, rehydrated through graded ethanol, and stained with hematoxylin (5 min) and eosin (3 min). After dehydration and mounting with neutral balsam, slides were imaged under a light microscope (Nikon, Japan). Inflammation score of lung tissues was performed by two blinded independent observers, according to criteria described by Ichimura [26].

Masson staining was performed using Masson’s trichrome stain (Solarbio, #G1340) to visualize collagen fibers (blue), muscle fibers (red), and cell nuclei (black) in lung sections. After staining, sections were rinsed, dehydrated, and mounted. Fibrotic areas were identified under a light microscope (Nikon, Japan).

### Immunohistochemistry (IHC) and multiplex immunohistochemistry (mIHC) analysis

For IgG detection in lung tissues, paraffin sections were deparaffinized, subjected to antigen retrieval, endogenous enzyme quenching, and blocked with 5% goat serum. Sections were then incubated at 4 °C overnight with anti-mouse IgG antibody (Abclonal, #3600000107). After washing, samples were incubated with Alexa Fluor 488-conjugated goat anti-mouse IgG secondary antibody for signal visualization. Nuclei were counterstained with DAPI.

For α-SMA or Ccl6 detection in lung tissues, sections were incubated overnight at 4 °C with primary antibodies against α-SMA (Proteintech, #14395-1-AP) or Ccl6 (Boster Biological Technology, #30384). After washing, sections were stained with HRP-conjugated secondary antibodies, followed by incubation with streptavidin–biotin complex (SABC) (BOSTER, #SA1022) for signal amplification. DAB was used for visualization, and nuclei were counterstained with hematoxylin. Slides were sealed and examined under a microscope (Nikon, Japan).

mIHC was performed on paraffin-embedded lung tissue sections to detect CD4^+^, CD8^+^ and CD11b^+^ cells simultaneously. Briefly, tissue sections were deparaffinized, antigen retrieval, inactivated endogenous enzymes and blocked with 5% goat serum. Sequentially, the sections were incubated with primary antibodies targeting CD4, CD8 and CD11b, and corresponding HRP-conjugated secondary antibodies. Tyramide-conjugated fluorophores (Servicebio, #G1226) were used for signal amplification to prevent cross-reactivity. Nuclei were counterstained with DAPI for cellular identification. Finally, fluorescence images were captured using a Zeiss Celldiscoverer 7 microscope (Carl Zeiss AG, Germany), and positive cell areas were quantified using ImageJ software.

All immunohistochemical and immunofluorescence-stained whole lung sections were digitally scanned at 20× magnification using a panoramic pathology scanner (PanoScanner 20, Panovue, China). Whole slide images were analyzed using HALO image analysis software (Indica Labs, Albuquerque, NM, USA). Quantification of positive cells was performed using “Multiplex IHC” and “HighPlex FL” module according to the manufacturer’s instructions, with consistent threshold settings applied across all samples.

### RNA-Seq analysis

Lung tissues were collected from four groups of TRIM21-immunized mice (*n* = 3 per group) at day 25 following poly(I:C) administration. Total RNA was extracted for bulk RNA sequencing, which was performed by Guangzhou Gidio Biotech Co., Ltd. (Guangzhou, China) using the NovaSeq 6000 platform (Illumina). Principal Component Analysis (PCA) was performed and visualized using the ggplot2 and PCAtools packages. Functional analysis of differentially expressed genes (DEGs)was conducted using the clusterProfiler package for Kyoto Encyclopedia of Genes and Genomes (KEGG) pathway enrichment and Gene Set Enrichment Analysis (GSEA). Volcano plots, heatmaps and enrichment plots were generated using ggplot2 and pheatmap packages.

### Western Blot (WB)

Left lung lobes were homogenized in RIPA buffer containing protease inhibitors using a homogenizer to ensure complete tissue disruption. After centrifugation at 12,000 × g for 10 min at 4 °C, the supernatant was collected for subsequent analysis. Protein concentration was determined using a bicinchoninic acid (BCA) protein assay kit (Vazyme, #E112) following the manufacturer’s protocol. Equal amounts of protein (30 µg) were separated by SDS-PAGE and transferred to PVDF membranes. The membranes were blocked with 5% non-fat milk and incubated overnight at 4 °C with primary antibodies against α-SMA (Proteintech, #14395-1-AP) and GAPDH (Proteintech, #60004-1-Ig). After washing, the membranes were incubated with HRP-conjugated secondary antibodies. Protein bands were detected using an enhanced chemiluminescence (ECL) detection system (NCM Biotech, #P2300), and visualized in a ChemiDoc MP Imaging System (Bio-Rad, USA).

### Statistical analysis

Statistical analyses were conducted using GraphPad Prism (San Diego, CA, USA) and R software. For each dataset, normality was first assessed using the Shapiro–Wilk test. For normally distributed data, comparisons between two groups were performed using Student’s t-test, and comparisons involving three or more groups were analyzed using one-way ANOVA followed by Tukey’s post hoc test. For data that did not meet normality assumptions (including cell proportions and antibody titers), the nonparametric Mann–Whitney U test (for two groups) or Kruskal-Wallis test followed by Dunn’s post hoc test (for multiple groups) was applied. Continuous data are presented as mean ± SEM.

## Results

### Characterization of systemic immune responses and localized lung changes following TRIM21 immunization

**Fig. 1:**
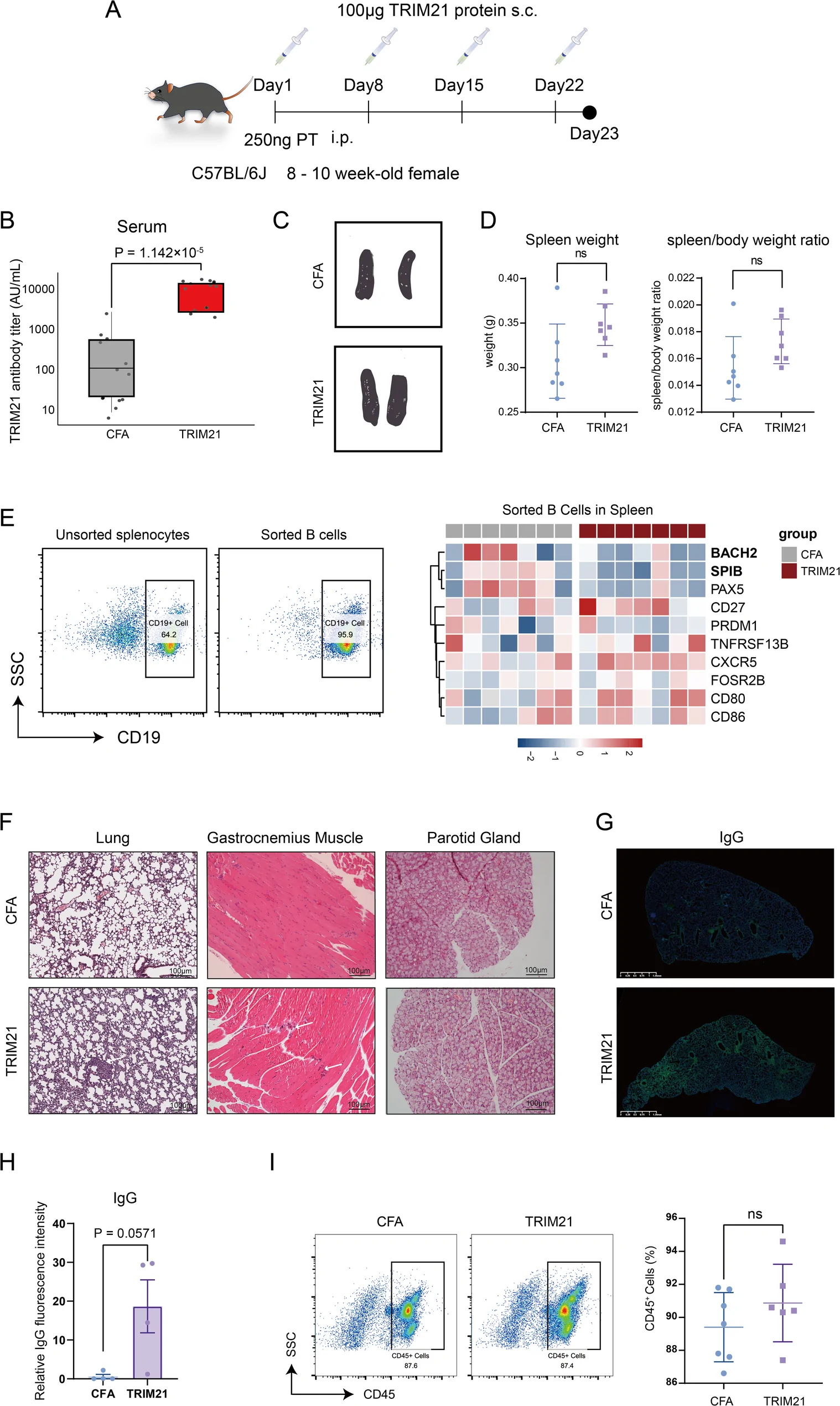
Establishment and validation of the TRIM21-autoimmunized model. **A** Immunization scheme. Mice received subcutaneous (s.c.) injections of recombinant TRIM21 emulsified in complete freund’s adjuvant (CFA) on days 1, 8, 15, and 22, with intraperitoneal (i.p.) pertussis toxin (PT) administration. Controls received CFA/PT only. **B** Serum anti-TRIM21 autoantibody titers detected by ELISA using 5-fold serial dilutions. **C**, **D** Spleen morphology and metrics, including representative spleen morphology images (**C**) and absolute weight and spleen-to-body weight ratio (**D**) (*n* = 7 mice per group). **E** Spleen B cell sorting and mRNA expression. CD19⁺ purity was analyzed by flow cytometry (left), and transcript levels of B cell activation markers were detected by real-time PCR (right) (*n* = 7 mice per group). **F** H&E staining of lung, muscle, and parotid gland (arrows indicate infiltrates). **G**, **H** Immunofluorescence staining of IgG deposition (green) in lung tissue sections (**G**) and quantification of IgG deposition in whole lung lobes (**H**) (*n* = 4 mice per group). **I** Flow cytometric analysis of lung CD45^+^ leukocyte. Data are presented as means ± SEM (bars) or min to max (box plots). *P* < 0.05; ns, not significant, analyzed by Student’s t-test. CFA, Immunization with CFA-emulsified control solution; TRIM21, Immunization with CFA-emulsified TRIM21 protein; ELISA, enzyme-linked immunosorbent assay; H&E, hematoxylin and eosin; IgG, immunoglobulin G

We first established a murine model of TRIM21 autoimmunity by immunizing mice with recombinant full-length murine TRIM21 protein emulsified in CFA (Fig. 1A). Protein integrity was confirmed by SDS-PAGE, showing a distinct band at ~ 70 kDa (Fig. S1). Over 60% of immunized mice developed high-titer anti-TRIM21 antibodies (≥ 1:15625), whereas all CFA controls had titers < 1:625 (*p* = 1.142 × 10^− 5^, Fig. 1B, Fig. S2), confirming robust autoantibody induction.

Although spleen weight and spleen/body weight ratios were mildly increased in TRIM21-immunized mice, the differences did not reach statistical significance (Fig. 1C-D). Transcriptomic profiling of sorted splenic B cells revealed trends toward downregulation of Spi-B Transcription Factor (SPIB) (*p* = 0.038, Fig. S3A) and BACH Transcriptional Regulator 2 (BACH2) (*p* = 0.097, Fig. S3B), which are involved in B-cell differentiation (Fig. 1E) [27, 28].

Histological examination revealed varying degrees of localized perivascular immune cell clusters in the lung tissues of immunized mice, whereas skeletal muscle showed only scattered inflammation infiltrates without necrosis, and salivary glands appeared histologically normal (Fig. 1F, Fig. S4). To further assess humoral immune involvement in the lung, we performed immunofluorescence staining for IgG in two groups. As shown in Fig. 1G, immunofluorescence analysis revealed differential patterns of IgG deposition in the lung tissue between the groups. Quantitative analysis of lung lobes showed a trend toward increased perivascular IgG deposition in TRIM21-immunized mice compared to CFA controls, although this did not reach conventional significance (Fig. 1H, *p* = 0.0571). Consistent with this localized pattern, flow cytometric analysis of lung single-cell suspensions showed no significant increase in the overall percentage of CD45^+^ immune cells (Fig. 1I). These findings suggest that systemic TRIM21 autoimmunity only induces a baseline state of subtle, localized pulmonary immune engagement rather than widespread inflammation.

### Impact of poly(I:C) exposure on the pulmonary environment in TRIM21-immunized mice

**Fig. 2:**
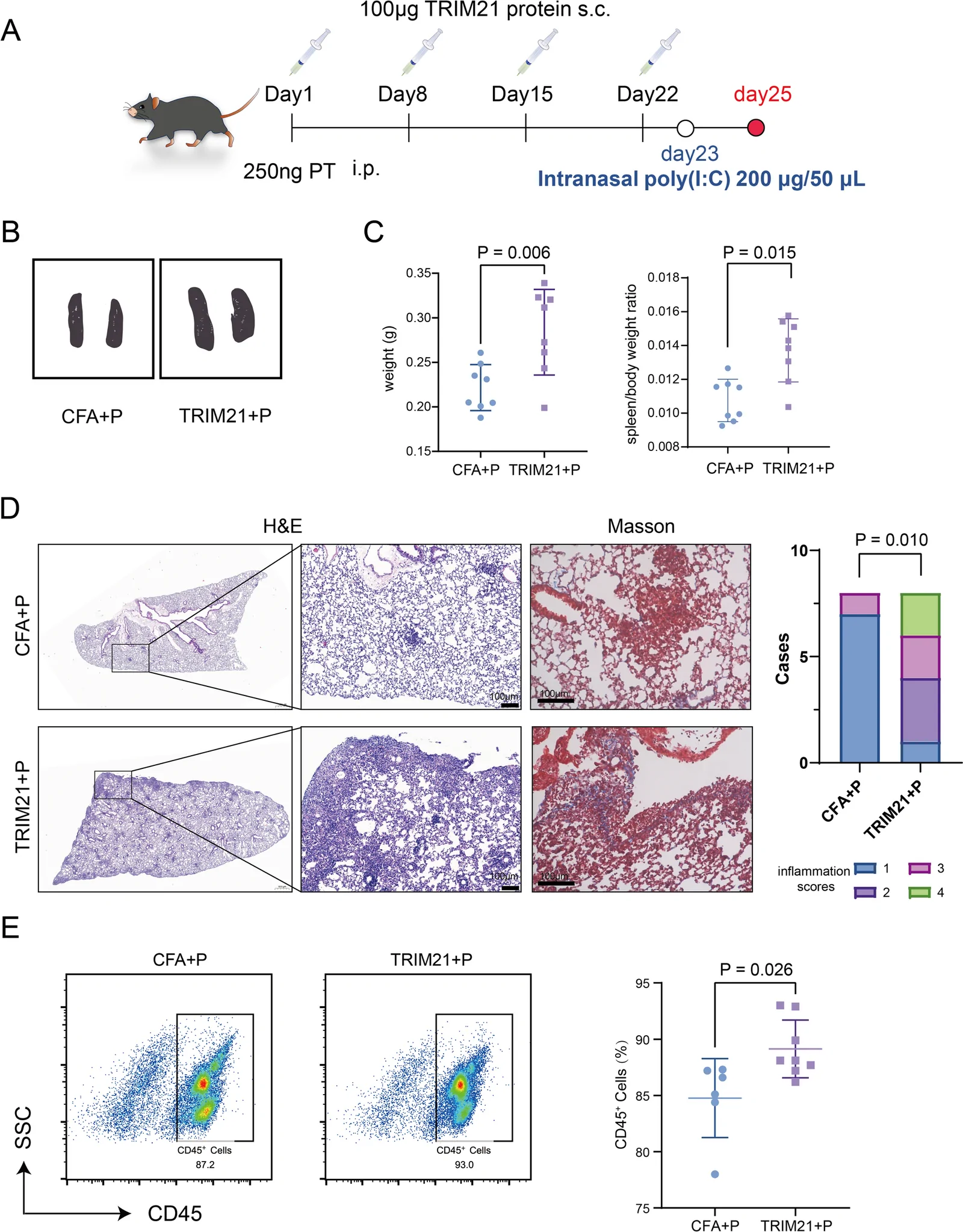
Poly(I:C) exacerbates pulmonary pathology in TRIM21-autoimmunized mouse model. **A** Experimental timeline for poly(I:C) boost administration scheme. **B**, **C** Spleen morphology and metrics, including representative spleen morphology images (**B**), spleen weight and spleen-to-body weight ratio in TRIM21 + P vs. CFA + P mice (**C**). **D** Representative images of lung histopathology. H&E (inflammation) and Masson’s trichrome (collagen deposition; blue). Semiquantitative analysis of inflammation scores is shown at the bottom (*n* = 8 mice per group). **E** CD45⁺ leukocyte frequency in lung tissues determined by flow cytometry. All data are presented as mean ± SEM (*n* = 7–8 mice per group). Statistical comparisons were performed using Student’s t test or Mann–Whitney’s U test. CFA + P, immunization with CFA-emulsified control solution plus supplemental poly(I:C); TRIM21 + P, immunization with CFA-emulsified TRIM21 protein plus supplemental poly(I:C); H&E, hematoxylin and eosin

To mimic viral infection and assess the impact of innate immune activation, TRIM21-immunized mice were administered intranasal poly(I:C) as a second-hit stimulus (TRIM21 + poly(I:C)) (Fig. 2A). Compared to poly(I:C)-only controls, TRIM21 mice showed significantly increased spleen weight (0.28 ± 0.048 g vs. 0.22 ± 0.025 g, *p* = 0.006) and spleen/body weight ratio (*p* = 0.015), reflecting systemic immune activation (Fig. 2B-C).

Histological analysis of lung tissue using H&E staining revealed that TRIM21 + poly(I:C) mice exhibited more severe pulmonary pathology compared to CFA+poly(I:C) controls, characterized by dense inflammatory cell infiltration, widespread immune cell aggregation, alveolar collapse, and marked disrupted lung architecture. Histological scoring confirmed a significant shift toward higher severity in the TRIM21 + poly(I:C) group, whereas the CFA+poly(I:C) group predominantly exhibited lower scores (*p* = 0.010, Fig. 2D). Masson’s trichrome staining and α-SMA immunohistochemistry demonstrated focal collagen deposition in TRIM21 + poly(I:C) group (Fig. 2D, Fig. S5A). However, α-SMA protein levels remained unchanged by Western blot (Fig. S5B), suggesting that fibrosis was likely in an early or localized stage.

Notably, under poly(I:C) exposure, immune cell infiltration was increased in the lungs of autoimmune mice. Flow cytometry showed elevated CD45⁺ leukocyte frequencies in TRIM21 + poly(I:C) mice compared to controls (89.14 ± 2.57% vs. 84.77 ± 3.51%, *p* = 0.026, Fig. 2E). Together, these results provide evidence supporting a two-hit pathogenic model in which TRIM21 autoimmunity may acts as a predisposing factor that primes the pulmonary immune niche, and poly(I:C) serves as a potent amplifier that enhances inflammation, promotes leukocyte infiltration, and initiates fibrotic remodeling. This dynamic closely parallels clinical observations in CTD-ILD, where viral triggers often precipitate acute lung injury.

### Immune cell infiltration patterns in TRIM21 autoimmunity

**Fig. 3:**
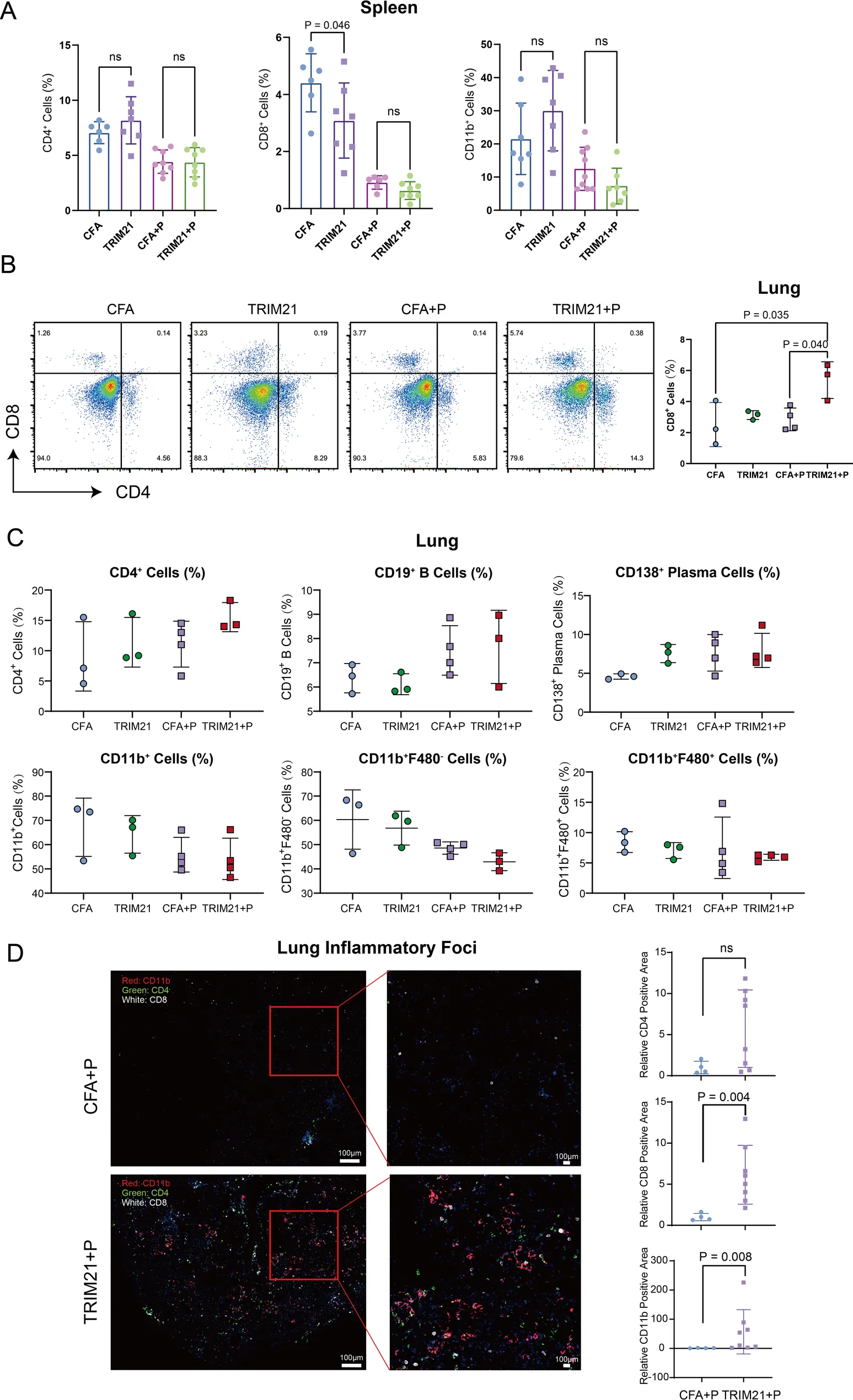
Immune profiling in spleen and lung tissues with Poly(I:C) enhancement. **A** Flow cytometric analysis for splenic immune subsets (CD4⁺, CD8⁺, and CD11b⁺ myeloid cells). **B** Flow cytometric analysis for lung CD8⁺ cells. Left: Representative gating strategy. Right: Quantification across groups. **C** Flow cytometric analysis for lung frequencies of CD4⁺, CD19⁺, CD138⁺, CD11b⁺ cells, macrophages (CD11b⁺F4/80⁺), and CD11b⁺F4/80⁻ cells. **D** Multiplex IHC for CD11b⁺ (red), CD4⁺ (green), CD8⁺ (white) cells in lung. Representative images and quantified analysis for positive areas are shown. All data are presented as mean ± SEM (*n* = 3–4 mice per group). Statistical comparisons were performed using Student’s t-test or one-way ANOVA with Tukey’s post-hoc test. CFA, immunization with CFA-emulsified control solution; TRIM21, immunization with CFA-emulsified TRIM21 protein. CFA + P, immunization with CFA-emulsified control solution plus supplemental poly(I:C); TRIM21 + P, immunization with CFA-emulsified TRIM21 protein plus supplemental poly(I:C); IHC, immunohistochemistry

To elucidate the cellular basis of TRIM21-mediated lung inflammation, we next analyzed immune cell dynamics by flow cytometry and immunofluorescence. A compartment-specific redistribution of CD8⁺ cells was observed. In the spleen, TRIM21-immunized mice exhibited a significant reduction in CD8⁺ cell frequency compared to CFA controls (3.086% vs. 4.407%, *p* = 0.046), with a numerical but non-significant trend following poly(I:C) boost (0.631% vs. 0.915%, *p* > 0.05, Fig. 3A, Fig. S6). These data suggest an alteration in the systemic T-cell compartment during the TRIM21 autoimmune response.

Conversely, in the lungs, the CD8^+^ cell frequency in TRIM21-immunized mice showed a mild upward trend (3.127% vs. 2.513%, *p >* 0.05). Furthermore, flow cytometric analysis of whole-organ suspensions revealed a modest significant increase in CD8⁺ cell frequency in TRIM21 + poly(I:C) mice compared to controls (5.380% vs. 2.850%, *p* = 0.040, Fig. 3B). Other immune subsets, including CD4⁺ cells and B cells, remained largely unchanged by flow cytometry (Fig. 3C).

Interestingly, while global quantitative analysis across the entire lung parenchyma revealed no statistically significant differences in overall immune cell densities (Fig. S7A-B), targeted multiplex immunofluorescence analysis of localized inflammatory lesions showed a striking spatial enrichment of specific effectors. Within these focal areas, we observed a 6.37-fold increase in the density of CD8⁺ cells (*p* = 0.004) and a 33.5-fold increase in CD11b⁺ myeloid cells (*p* = 0.008) compared with controls (Fig. 3D).

Together, these findings demonstrate that the two-hit model triggers focal, niche-specific recruitment of cytotoxic T cells and innate myeloid cells into the lung parenchyma. This spatially organized immune response represents a key feature of the amplified inflammation observed in our model, mirroring the patchy, interstitial nature of clinical CTD-ILD.

### Transcriptomic profiling reveals immune pathways associated with TRIM21-driven lung inflammation

**Fig. 4:**
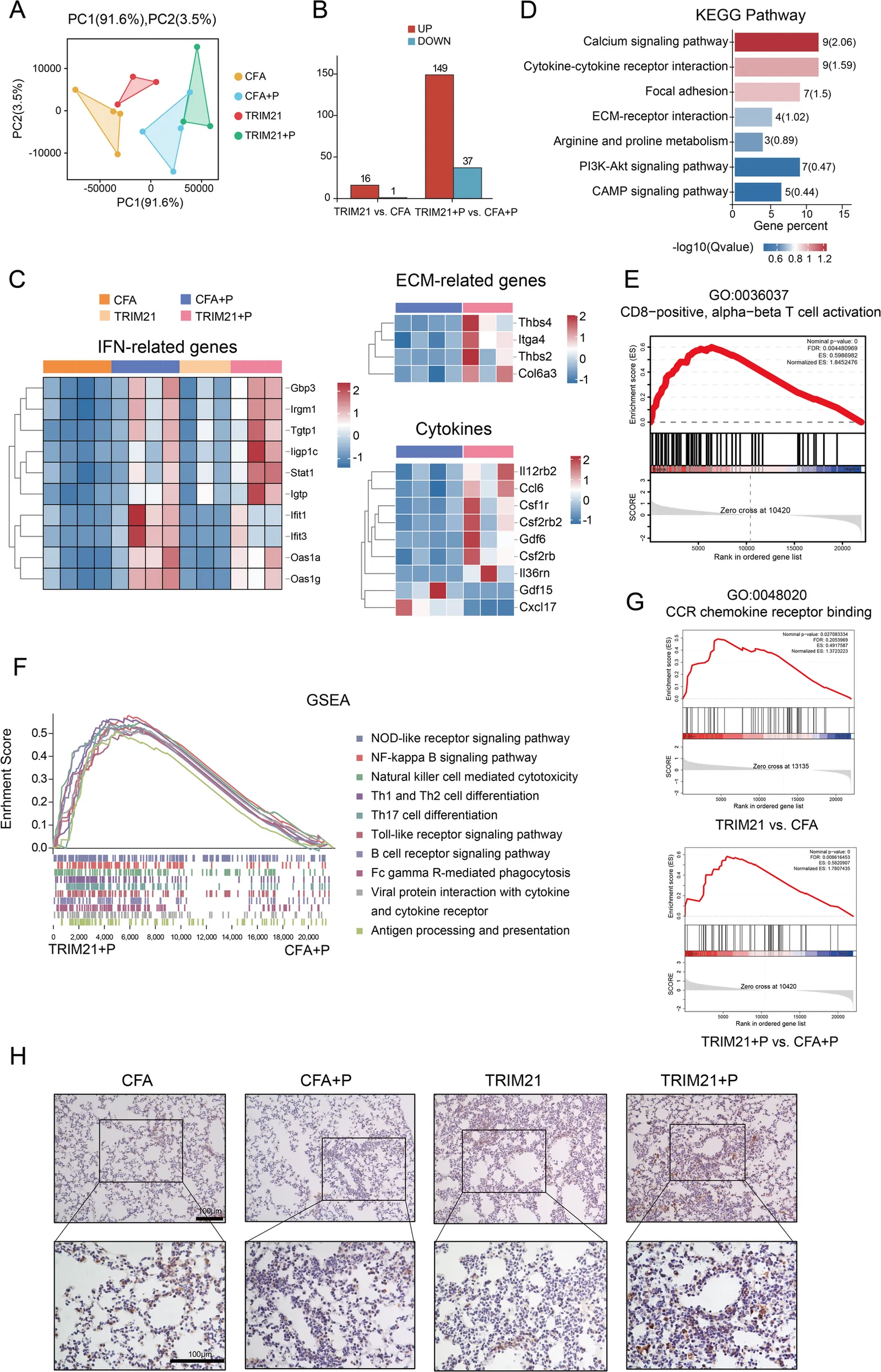
RNA-seq reveals distinct gene expression patterns in four groups of mice. **A** Principal component analysis showing group segregation. **B** Bar graph displaying differentially expressed genes (DEGs) between TRIM21 and CFA groups, as well as in Poly(I:C) treated mice. **C** Heatmap analysis showing IFN-related genes, cytokines, and extracellular matrix (ECM) regulators. **D** KEGG enrichment analysis of DEGs between TRIM21 + P and CFA + P groups. **E**, **F** GSEA of T cell activation and immune related pathways. **G** GSEA of CCR chemokine receptor signaling pathway enrichment in the presence or absence of poly(I:C) stimulation. **H** Immunohistochemical (IHC) detection of Ccl6 expression in mouse lung tissue sections. Representative images were shown. CFA, immunization with CFA-emulsified control solution; TRIM21, immunization with CFA-emulsified TRIM21 protein. CFA + P, immunization with CFA-emulsified control solution plus supplemental poly(I:C); TRIM21 + P, immunization with CFA-emulsified TRIM21 protein plus supplemental poly(I:C). DEG, differentially expressed gene; ECM, extracellular matrix; KEGG, Kyoto Encyclopedia of Genes and Genomes; GSEA, gene set enrichment analysis; IHC, immunohistochemistry

To explore the molecular mechanisms driving immune infiltration, we performed bulk RNA sequencing of lung tissues from all experimental groups. Principal component analysis (PCA) showed distinct clustering patterns according to different treatments, confirming global transcriptomic divergence induced by TRIM21 and poly(I:C) (Fig. 4A). TRIM21 immunization alone induced 17 DEGs compared to the CFA control, indicating only limited overall differences between the two groups, consistent with our histopathological findings. In contrast, poly(I:C) stimulation in TRIM21-immunized mice yielded 186 DEGs — an 11-fold increase relative to unstimulated TRIM21 mice (Fig. 4B, Fig. S8A-B), reflecting a strong synergy between autoimmunity and innate immune activation. Notably, several interferon-inducible genes were upregulated upon poly(I:C) stimulation, indicating engagement of antiviral and inflammatory signaling pathways and confirming the effectiveness of poly(I:C) induction (Fig. 4C). In contrast, we did not detect a clear effect of TRIM21 immunization on IFN-related gene expression, suggesting that TRIM21 may promote lung inflammation through IFN-independent mechanisms rather than direct interferon pathway activation. KEGG enrichment analysis revealed upregulation of pathways related to calcium signaling (Thbs2, Thbs4, Col6a3), cytokine–cytokine receptor interaction (Ccl6, Csf1r) and ECM-receptor interaction (Itga4) (Fig. 4C-D), collectively supporting a microenvironment favoring leukocyte recruitment and tissue remodeling.

Consistent with immune profiling, pathways related to CD8⁺ T cell activation, T cell differentiation, and proinflammatory responses were enriched in TRIM21 + poly(I:C) lungs (Fig. 4E-F). Gene Set Enrichment Analysis (GSEA) further identified significant enrichment of the “CCR chemokine receptor binding” pathway (NES = 1.78, FDR = 0.0086) (Fig. 4G), pointing to chemokine-mediated recruitment as a central mechanism. Given that Ccl6 emerged as the most significantly upregulated chemokine in our transcriptomic analysis and has been well documented for its role in recruiting macrophages and diverse immune cell subsets [29], we further validated its expression in lung tissue. Immunohistochemical staining revealed that TRIM21 + poly(I:C) mice displayed substantially increased percentages of Ccl6-positive cells in lung tissue compared to the other three groups (Fig. 4H, Fig. S9A-B). This was corroborated by qRT-PCR analysis, which showed significantly elevated Ccl6 mRNA expression in TRIM21 + poly(I:C) group relative to CFA+poly(I:C) group (Fig. S9C, *p* = 0.029). These findings indicate that TRIM21 autoimmunity synergizes with poly(I:C) stimulation to amplify Ccl6-mediated chemotactic signaling in the lungs.

Together, these data delineate a two-step pathogenic model: TRIM21-induced autoimmunity primes the lung microenvironment, and poly(I:C)-induced viral mimicry potentiates local immune activation through Ccl6-mediated recruitment of cytotoxic and myeloid populations. This inflammatory amplification loop culminates in pronounced CD8⁺ cell-driven pulmonary immune infiltration and tissue injury.

## Discussion

This study provides the first in vivo evidence that TRIM21 (Ro52) autoimmunity is associated with increased susceptibility to lung inflammation, suggesting a potential link between anti-Ro52 antibodies and CTD-ILD. Using a murine immunization model with full-length TRIM21 protein, we show that TRIM21 autoimmunity is associated with a trend toward enhanced pulmonary immune infiltration. Upon poly(I:C) challenge mimicking viral exposure, this immune response is markedly amplified, with enhanced CD8⁺ and CD11b⁺ cell infiltration and increased expression of the chemokine Ccl6, a murine ortholog of human CCL15 and CCL23. These findings indicate a potential two-hit pathogenic model, in which TRIM21 autoimmunity first predisposes the lung to an inflammatory baseline, and subsequent viral-like innate immune activation then exacerbates immune cell recruitment and tissue injury.

Our model was designed to recapitulate the immune-initiating and acute exacerbation phases of anti-Ro52 (TRIM21)–associated ILD rather than the chronic fibrotic stage typically observed in advanced CTD-ILD. Clinically, anti-Ro52 antibodies are frequently detected across connective tissue diseases—including ASyS, anti-MDA5⁺ DM, and SSc—and are consistently associated with increased ILD prevalence, greater disease severity, and adverse outcomes [10–13]. Notably, patients co-expressing anti-MDA5 and anti-Ro52 antibodies often develop a rapidly progressive ILD phenotype [11, 12], supporting a synergistic interplay between antiviral and autoimmune pathways. The present model is consistent with these clinical observations, illustrating how TRIM21 autoimmunity may contribute to a pre-inflammatory lung environment that becomes highly susceptible to secondary viral or viral-like triggers, especially in patients co-expressing anti-MDA5 antibodies.

A notable feature of our model is the prominent pulmonary involvement of the inflammatory response. Despite systemic immunization and robust circulating anti-TRIM21 autoantibodies, immune cell infiltration and IgG deposition were predominantly observed in pulmonary tissue, with minimal involvement of muscle or salivary glands. This tropism may reflect the unique immunological landscape of the lung—a highly vascularized, antigen-rich environment constantly exposed to airborne pathogens and equipped with a complex network of immune and non-immune cells, including alveolar macrophages, epithelial cells, and tissue-resident lymphocytes [30, 31]. Beyond these anatomical factors, the tissue-specific expression of the target antigen itself likely plays a decisive role, as TRIM21 is abundantly expressed in pulmonary tissue and acts as a key regulator of antiviral immunity [17, 20, 32]. In systemic lupus erythematosus (SLE), studies have shown that anti-TRIM21 autoantibodies are associated with suppressed TRIM21 protein expression, enhanced IFN-I production, and abnormal B cell differentiation [33]. Aligned with this clinical evidence, our findings suggest that these autoantibodies could potentially interfere with TRIM21’s endogenous immunoregulatory roles and promoting inflammation within the lung. This autoimmune targeting might compromise local tissue protection, rendering the lung particularly vulnerable to immune-mediated injury.

At the mechanistic level, characterization of the pulmonary immune landscape revealed a pronounced shift toward a cytotoxic and proinflammatory milieu. Flow cytometry and multiplex immunohistochemistry demonstrated selective enrichment of cytotoxic CD8⁺ cells and CD11b⁺ myeloid cells, whereas CD4⁺ cells and B cells exhibited almost no changes. Notably, TRIM21 immunization alone reduced splenic CD8^+^ cells without a significant concomitant increase in the lung. However, subsequent poly(I:C) challenge drove a marked accumulation of CD8^+^ cells in the pulmonary tissue. This distinct dynamic suggests that while TRIM21 autoimmunity mobilizes the systemic CD8^+^ cell pool, a secondary viral-like trigger is required to actively recruit these cells into the lung, resulting in a localized inflammatory response exacerbated by pre-existing TRIM21 autoimmunity. These results are consistent with the recent studies that identifying a pathogenic role for lung-resident or clonally expanded CD8⁺ T cells in CTD-ILD [34]. Transcriptomic profiling confirmed activation of cytotoxic and chemokine pathways, most prominently Ccl6, a rodent ortholog of human CCL15/CCL23, known to recruit macrophages and cytotoxic lymphocytes. These findings support a model in which TRIM21 autoimmunity creates a chemokine-rich, proinflammatory niche that promotes CD8⁺ cell and myeloid cell recruitment or expansion of cytotoxic and myeloid cells, amplifying tissue damage. Although CD4⁺ cells and humoral immunity likely contribute to this milieu, CD8⁺ cytotoxic effector activity appears to be the principal driver of lung injury.

Our findings also highlight the critical role of viral-like stimuli in amplifying TRIM21-mediated pathology. Poly(I:C), a synthetic analog of double-stranded viral RNA, significantly worsened lung inflammation in TRIM21-immunized mice, recapitulating clinical observations that respiratory infections often precede ILD exacerbations in CTDs [35]. While previous studies have proposed that viral triggers may initiate TRIM21 autoimmunity [36], our data further suggest that such triggers also act as secondary amplifiers of immune-mediated tissue injury. This supports a two-hit model in which viral mimicry synergizes with preexisting TRIM21 autoimmunity to intensify lung inflammation.

This study has several important implications. Scientifically, it supports a pathogenic contribution of a direct pathogenic link between TRIM21 autoimmunity and lung inflammation, moving beyond the view of anti-Ro52 as a passive biomarker. The identification of Ccl6 as a key chemokine involved in CD8⁺ T cell recruitment offers a novel molecular target for therapeutic intervention. Clinically, our results support precision medicine strategies for Ro52-positive ILD, including blockade of chemokine pathways (e.g., the CCL6–CCR1/CCR3 axis) or modulation of CD8⁺ T cell cytotoxicity. Moreover, the two-hit model reinforces the importance of vigilant infection control in these patients, especially during immunosuppressive treatment or viral outbreaks.

Our study also provides mechanistic insight into prior clinical observations. In our previous multicenter cohort, co-positivity for anti-MDA5 and anti-Ro52 defined a particularly high-risk subset of DM patients with significantly increased mortality, especially in those with RP-ILD [11]. The present study may support this synergy: TRIM21 autoimmunity may modulate the activation state, differentiation trajectory, or responsiveness of systemic CD8^+^ T cells, thereby lowering the threshold for tissue-specific inflammatory responses. These responses are subsequently exacerbated by MDA5 activation and viral-like innate signals, culminating in severe lung injury.

Despite its strengths, our study has several limitations. First and foremost, we must explicitly state that the current findings represent an observational association rather than a demonstration of absolute causality. While our model effectively recapitulates the clinical reality where anti-Ro52 antibodies serve as a harbinger of severe lung disease, it does not yet define the specific effector mechanism. Because passive antibody transfer and antigen-specific T-cell functional assays were not performed, we cannot definitively exclude the potential influence of the broader inflammatory milieu induced by the immunization protocol. Consequently, our conclusions are calibrated to view TRIM21 autoimmunity as a ‘priming factor’ that establishes a pro-inflammatory pulmonary niche. To further dissect this relationship, future studies employing TRIM21-deficient models and adoptive transfer experiments are warranted to isolate the independent pathogenic roles of humoral versus cellular components of the TRIM21-specific immune response. Second, while our current model recapitulates the early inflammatory phase of TRIM21-associated lung disease, it does not fully capture the chronic fibrotic stage observed in human CTD-ILD. Longitudinal and repetitive-challenge studies are warranted to determine whether acute inflammation resolves or progresses to fibrosis and to identify therapeutic windows for intervention. Furthermore, future studies incorporating systematic multi-organ histological and quantitative analyses will be necessary to fully determine the extent and relative specificity of systemic organ involvement in this model. In addition, our findings regarding CD8⁺ cells and CCL6 require further investigation in both mouse models and patients, as well as additional functional studies and clinical correlation analyses in anti-Ro52-positive ILD patients. Finally, it is important to note that our preliminary flow cytometry panel included CD4 and CD8 markers without CD3 gating or CD11c exclusion. Therefore, although the identified CD8⁺ and CD4⁺ populations are most likely predominantly T lymphocytes, the potential inclusion of minor subsets, such as CD8⁺ dendritic cells or NKT cells, cannot be completely ruled out. Future studies employing comprehensive multi-color panels are warranted to definitively characterize these specific T-cell lineages.

## Conclusions

Taken together, our findings suggest that anti-Ro52 is not merely a prognostic marker but may also play a pathogenic role in ILD. TRIM21 autoimmunity primes the lung for inflammatory responses, including CD8⁺ cell-associated inflammation, which are amplified by viral-like innate stimuli—supporting a “two-hit” model of disease. Recognizing this mechanism may enable more precise risk stratification and guide targeted therapies, such as cytotoxic T lymphocyte modulation or CCL6 blockade.

## Supplementary Information

Additional file 1: Figure S1. TRIM21 protein validation. Coomassie Brilliant Blue (CBB)-stained SDS-PAGE.

Additional file 2: Figure S2. Serum anti-TRIM21 antibody titers were determined by ELISA. CFA, immunization with CFA-emulsified control solution; TRIM21, immunization with CFA-emulsified TRIM21 protein.

Additional file 3: Figure S3. Gene expression analysis of splenic B cells in TRIM21 autoimmune model. Quantitative RT-PCR analysis of Spib (A) and Bach2 (B) in sorted splenic B cells from TRIM21-immunized and control mice (n = 6 mice per group). Gene expression normalized to β-actin. Data presented as mean ± SEM. Statistical significance was determined by Mann-Whitney U test; P values are indicated on the graphs.

Additional file 4: Figure S4. Representative H&E staining of lung tissue in CFA and TRIM21 groups. Representative hematoxylin and eosin (H&E) staining images of lung tissue from CFA and TRIM21 groups (*n* = 4 mice per group). CFA, immunization with CFA-emulsified control solution; TRIM21, immunization with CFA-emulsified TRIM21 protein; H&E, hematoxylin and eosin.

Additional file 5: Figure S5. Assessment of α-Smooth muscle actin (α-SMA) expression in lung tissues. Representative images of immunohistochemistry (IHC) staining (A) and western blot analysis (B). CFA, immunization with CFA-emulsified control solution; TRIM21, immunization with CFA-emulsified TRIM21 protein. CFA + P, immunization with CFA-emulsified control solution plus supplemental poly(I:C); TRIM21 + P, immunization with CFA-emulsified TRIM21 protein plus supplemental poly(I:C); α-SMA, alpha-smooth muscle actin.

Additional file 6: Figure S6. Flow cytometry gating strategy for detection of T cell subtypes. Live single cells were gated to identify CD4+ and CD8+ cell populations.

Additional file 7: Figure S7. Multiplex immunofluorescence analysis of immune cell infiltration in lung tissue. (A) Representative multiplex immunofluorescence staining of lung sections from CFA + poly(I:C) and TRIM21 + poly(I:C) mice. CD4 (green), CD8 (orange), CD11b (yellow), and DAPI (blue). Right panels show magnified views with white arrowheads indicating positively stained cells. (B) Quantification of CD4+, CD8+, and CD11b+ cells in whole lung lobes. Data are presented as mean ± SEM (n = 3 mice per group).

Additional file 8: Figure S8. Transcriptional consequences of poly(I:C) challenge. (A) Venn diagram of overlapping differentially expressed genes (DEGs) between comparisons: TRIM21 vs. CFA control, and TRIM21 + P vs. CFA + P. (B) Validation of poly(I:C) bioactivity. Volcano plot showing transcriptional changes in CFA + P vs. CFA controls. CFA, immunization with CFA-emulsified control solution; TRIM21, immunization with CFA-emulsified TRIM21 protein. CFA + P, immunization with CFA-emulsified control solution plus supplemental poly(I:C); TRIM21 + P, immunization with CFA-emulsified TRIM21 protein plus supplemental poly(I:C).

Additional file 9: Table S1. Primers for quantitative real-time PCR.

## Data Availability

Data will be made available on request.

## Abbreviations

ILD: Interstitial lung disease
CTD: Connective tissue diseases
IIM: Inflammatory myopathies
SSc: Systemic sclerosis
pSS: Primary Sjögren’s syndrome
CTD-ILD: CTD-associated ILD
TRIM21: Tripartite motif-containing protein 21
ASyS: Antisynthetase syndrome
DM: Dermatomyositis
RP-ILD: Rapidly progressive ILD
IFN-I: Type I interferon
SLE: Systemic lupus erythematosus
EBV: Epstein–Barr virus
poly(I:C): Polyinosinic: polycytidylic acid
TLR3: Toll-like receptor 3
CFA: Complete Freund adjuvant
MACS: Magnetic-activated cell sorting
qRT-PCR: Quantitative real-time PCR
PFA: Paraformaldehyde
IHC: Immunohistochemistry
mIHC: Multiplex immunohistochemistry
DAB: 3,3’-diaminobenzidine
SABC: Streptavidin–biotin complex
KEGG: Kyoto Encyclopedia of Genes and Genomes
GSEA: Gene Set Enrichment Analysis
WB: Western Blot
BCA: Bicinchoninic acid
BACH2: BACH Transcriptional Regulator 2
SPIB: Spi-B Transcription Factor
